# Cross-sectional associations between personality traits and device-based measures of step count and sedentary behaviour in older age: the Lothian Birth Cohort 1936

**DOI:** 10.1186/s12877-019-1328-3

**Published:** 2019-11-10

**Authors:** Iva Čukić, Catharine R. Gale, Sebastien F. M. Chastin, Philippa M. Dall, Manon L. Dontje, Dawn A. Skelton, Ian J. Deary, on behalf of the Seniors USP Team, Dawn A. Skelton, Sebastien Chastin, Simon Cox, Elaine Coulter, Iva Čukić, Philippa Dall, Ian Deary, Geoff Der, Manon Dontje, Claire Fitzsimons, Catharine Gale, Jason Gill, Malcolm Granat, Cindy Gray, Carolyn Greig, Elaine Hindle, Karen Laird, Gillian Mead, Nanette Mutrie, Victoria Palmer, Ratko Radaković, Naveed Sattar, Richard Shaw, John Starr, Sally Stewart, Sally Wyke

**Affiliations:** 1 Department of Psychology, Centre for Cognitive Ageing and Cognitive Epidemiology, University of Edinburgh, Edinburgh, UK; 2 Centre for Dementia Prevention, University of Edinburgh, Edinburgh, UK; 3 MRC Lifecourse Epidemiology Unit, University of Southampton, Southampton, UK; 4 Centre for Living, School of Health and Life Sciences, Glasgow Caledonian University, Glasgow, UK; 5 Department of Movement and Sports Sciences, Faculty of Medicine and Health Science, Ghent University, Ghent, Belgium; 6 Department of Public Health, Erasmus MC, University Medical Centre Rotterdam, Rotterdam, The Netherlands

**Keywords:** Device-based measures, Physical activity, Sedentary behaviour, activPAL, Personality traits, Older age

## Abstract

**Background:**

While the associations between personality traits and self-reported physical activity are well replicated, few studies have examined the associations between personality and device-based measures of both physical activity and sedentary behaviour. Low levels of physical activity and high levels of sedentary behaviour are known risk factors for poorer health outcomes in older age.

**Methods:**

We used device-based measures of physical activity and sedentary behaviour recorded over 7 days in 271 79-year-old participants of the Lothian Birth Cohort 1936. Linear regression models were used to assess whether personality traits were cross-sectionally associated with step count, sedentary time, and the number of sit-to-stand transitions. Personality traits were entered one at a time, and all-together, controlling for age and sex in Model 1 and additionally for BMI and limiting long-term illness in Model 2.

**Results:**

None of the associations between personality traits and measures of physical activity and sedentary behaviours remained significant after controlling for multiple-comparisons using the False Discovery Rate test (all *p*s > .07).

**Conclusions:**

We found no evidence that personality traits are associated with device-based measures of physical activity or sedentary behaviour in older age. More studies are needed to replicate and examine the nature of these relationships.

## Background

Physical inactivity is a well-recognised risk factor for poorer health in older age. In addition, sedentary behaviour, defined as any waking activity in a sitting or reclining posture characterised by low energy expenditure (≤ 1.5 metabolic equivalents (METs)), [1, 2] has been identified as a risk factor for a wide range of adverse health outcomes, [3–6] including all-cause mortality [7]. It may be independent of, or attenuated by physical activity [8]. Older adults are the least physically active and the most sedentary portion of the population [9, 10]. It is, therefore, of particular importance to understand patterns of sedentary and physical activity behaviour in older adults, due to their potential health costs to ageing populations worldwide [11].

Well-known correlates of self reported physical activity are personality traits of the Five Factor Model (FFM) [12, 13]. Personality traits are stable characteristics of ways of behaving and thinking, although several different models exist, the commonly used FFM has five primary traits, neuroticism (the tendency to experience negative emotions), extraversion (the tendency to be sociable and outgoing), openness (the tendency to be creative and unconventional), agreeableness (the tendency to be trusting and modest), and conscientiousness (the tendency to be disciplined and organised). The most recent meta-analysis of 64 studies and a total of 88,400 participants found that higher extraversion (*r* = .11), conscientiousness (*r* = .10), and openness (*r* = .03) were all related to higher levels of physical activity, whereas higher levels of neuroticism were related to lower levels of physical activity (*r* = −.07) [14]. The meta-analytic effect was not significant for agreeableness. Furthermore, a recent study of 339 twin pairs suggested that observed phenotypic associations between extraversion and neuroticism, and self-reported physical activity, may in part be due to overlapping genetic influences [15].

However, most previous studies utilised self-reported measures of physical activity, that may not be sufficiently accurate, [16] and that show only moderate associations with device-based measures [17]. One study used an accelerometer (no make provided) to record step count and MET levels in obese individuals in an intervention programme, and found that women (but not men) lower in neuroticism had overall lower levels of physical activity [18]. Another study examined personality correlates of device-based recordings (NL-1000 accelerometer) of physical activity in college women, [19] and found that higher neuroticism was related to lower levels of physical activity. The only study to date that examined the associations between personality traits and both device-based measures of physical activity and sedentary behaviour in older age was conducted by Artese et al., [20] utilising a 7-day continuous recording of an ActiGraph monitor. The study found that higher extraversion, agreeableness, and conscientiousness, as well as lower neuroticism were associated with being more engaged in moderate physical activity and having a higher step count. None of the personality traits were associated with the device-based measure of sedentary behaviour. However, the sample size of that study was small (*N* = 69) and thus likely to be underpowered, and the ActiGraph does not provide a measure of postural sitting but of lack of movement.

Understanding the associations of potential determinants such as personality traits with physical activity and sedentary behaviour can allow appropriate targeting of public health interventions. However previous research exploring the association of the five-factor model of personality traits with device-based measures of physical activity and sedentary behaviour, has either not been explored in older adults, or has been limited by small sample sizes and the lack of a postural measure of sedentary behaviour. The association of the pattern of accumulation of sedentary behaviour with personality traits has not been previously reported. Therefore, the aim of the current study was to examine cross-sectional associations between personality traits of the FFM, and device-based measures of physical activity and postural sedentary behaviour in a larger sample of older participants.

## Methods

### Participants

Participants for the Seniors USP study (Understanding Sedentary Patterns) were recruited from Wave 4 of the Lothian Birth Cohort 1936 (LBC1936) [21, 22] when they were about 79 years old. In total, 373 participants who attended Wave 4 for of the LBC1936 were asked to take part until 304 agreed and had the activPAL monitor fitted. Of those, two did not return a monitor, seven had incomplete sleep diaries, 20 did not have a full 7 days of activPAL data, and a further four were excluded due to insufficient quality of activPAL data. The sample included in the analysis, therefore, comprises 271 participants (131 females). Participants provided written informed consent, and ethical approval was obtained from the Multi-Centre Research Ethics Committee for Scotland and Lothian Research Ethics Committee.

### Measures

#### Physical activity/sedentary behaviour

Sedentary behaviour and physical activity were recorded continuously over 7 days (7x24h, starting at midnight) using the activPAL activity monitor, a tri-axial inclinometor that continuously monitors the position of the leg (activPAL3c, PAL Technologies Ltd., Glasgow, UK). The monitor is a small and light device (53x35x7mm; 15 g) that participants wore on the front of the thigh of their dominant leg, attached with a double-sides hypoallergenic sticky pad and covered with a waterproofing dressing. It is a well-validated and reliable method of recording sedentary time [23–25]. The participants wore the monitor for seven full days including during sleep, bathing or swimming. During that time, participants also kept a diary of the times they fell asleep and woke up each morning and night. Inclinometer data were downloaded using activPAL software version 7.2.32 (PAL Technologies Ltd., Glasgow, UK), and merged with sleep diary data using the statistical programming language R [26]. Three outcome measures were used. The average percentage of waking time spent sedentary per day (sedentary time) represented the total time spent in sedentary behaviour. The average number of sit-to-stand transitions was used to explore the pattern of sedentary behaviour. A sit-to-stand transition represents the start of a period of standing and/or stepping and a break in sedentary behaviour. This outcome measure, therefore, represented the total number of sedentary breaks each day, providing an indication of the pattern of accumulation of total sedentary behaviour. Finally, the average number of steps per day (step count) represented total physical activity. The number of sit-to-stand transitions and the step count variables were positively skewed, so square-root transformed variables were used in the analyses.

#### Personality traits

Personality was assessed using the 50-item International Personality Item Pool (IPIP) questionnaire, [27] that taps into the dimensions of the Five Factor Model (FFM) [28]. Participants rated 50 statements with regard to how well they described themselves (e.g. “I am the life of the party”) on a 5-point Likert scale from very inaccurate to very accurate, with some items scored positively and some reversed. Dimensions are scored as the sum of statements (reversed as required) relating to that dimension (10 statements per dimension). The IPIP dimensions are Extraversion, Conscientiousness, Agreeableness, Emotional Stability (reflecting reversely scored Neuroticism), and Intellect/Imagination (similar to Openness). The scale is a reliable and validated measure of the FFM dimensions [29].

#### Covariates

Personality traits, physical activity and sedentary behaviour have all been show to vary by age, gender, and health (including weight status), and the choice of covariates was made based on the recent research linking personality traits and device-based measures of physical activity [20]. Age at personality assessment was expressed in days, and standardised in the models for ease of interpretation. Sex was coded as 1 for men and 2 for women. BMI was entered as a continuous variable. Limiting long-term illness (hereafter long-term illness) was coded as 1 if the participant answered “yes “to both of the following questions: “Do you have any long-standing illness, disability or infirmity? “and “Does this condition limit your activities in any way? “and 0 if they answered “no” to either of the two questions.

### Statistical analysis

Males and females were compared using t-tests for continuous, and Chi-square tests for categorical variables. Linear regression was used to assess the cross-sectional associations between personality traits and physical activity/sedentary behaviour outcomes. Due to significant correlations between personality traits, we constructed regression models with each of the personality traits separately in addition to those where all traits are entered together [30]. Two models were fitted for each combination of personality trait(s) and each of the three outcomes: sedentary time, sit-to-stand transitions, and step-count. Model 1 controlled for age and sex. Model 2 was the same as Model 1, and additionally controlled for BMI, and long-term illness. Due to the large number of tests conducted, we controlled for multiple testing using the False Discovery Rate test [31]. All analyses were conducted using R version 3.3.1 [26].

## Results

### Descriptive statistics

Descriptive statistics for all variables in the study are presented in Table 1. Men were slightly more sedentary than women (approximately 65% of their waking time spent sedentary versus approximately 60% for women). They also had slightly lower agreeableness scores. There were no significant differences between the sexes in any other variables in the study. The matrix of correlations between the key variables in the study is presented in Table 2.

**Table 1:** Descriptive statistics of all variables in the study

	Males	Females	*P*-value for difference	Total
	*n* = 140	*n* = 131		*n* = 271
	M (SD)	M (SD)		M (SD)
Step count	6908.71 (2806.90)	6973.95 (2812.77)	.849	6940.25 (2804.72)
Sedentary time (%)	64.78 (9.79)	60.09 (10.48)	< .001	62.51 (10.38)
Sit-to-stand transitions	43.82 (12.46)	44.14 (10.40)	.816	43.97 (11.49)
BMI (kg/m^2^)	27.62 (4.06)	26.80 (4.51)	.115	27.22 (4.30)
Extraversion	20.44 (7.58)	22.05 (7.53)	.083	21.23 (7.59)
Agreeableness	29.16 (5.63)	33.20 (4.17)	< .001	31.15 (5.35)
Conscientiousness	28.01 (5.68)	27.63 (6.45)	.606	27.83 (6.06)
Emotional Stability	25.60 (7.28)	24.95 (7.19)	.463	25.28 (7.23)
Intellect/Imagination	23.96 (6.22)	23.96 (6.48)	.994	23.96 (6.34)
	*n*(%)	*n*(%)		*n*(%)
Long-term illness	24 (17.1)	25 (19.1)	.797	49 (18.1)

*Note.* Step count, sedentary time and sit-to-stand transitions are expressed as daily averages. Personality traits are given in raw units. *BMI* Body Mass Index

**Table 2:** Zero-order correlations between the measures of physical activity/sedentary behaviour, and each of the personality traits

	Step count	Sedentary Time	STS transitions	Extraversion	Agreeableness	Conscientiousness	Emotional Stability	Intellect
Step count	1.00							
Sedentary Time	− 0.48^***^	1.00						
STS transitions	0.25^***^	−0.02	1.00					
Extraversion	0.08	0.09	0.12	1.00				
Agreeableness	0.09	− 0.18^**^	− 0.02	0.36^***^	1.00			
Conscientiousness	0.12	−0.13^*^	0.01	0.10	0.25^***^	1.00		
Emotional Stability	0.09	0.02	−0.10	0.21^***^	0.24^***^	0.22^***^	1.00	
Intellect	0.04	−0.05	0.05	0.47^***^	0.33^***^	0.24^***^	0.12^*^	1.00

*Note.* STS transitions = Sit-to-stand transitions; ^*^p < .05; ^**^p < .01, ^***^p < .001

### Personality traits and step count

We first examined the associations between personality traits and the average daily number of steps. The first set of models included one personality trait at the time, controlling for age and sex, and an additional model with all five personality traits entered together (Model 1, Table 3). None of the personality traits was significantly associated with step-count. Model 2 (Table 3) further adjusted for BMI and long-term illness. Personality traits were again not significantly associated with step count. BMI and long-term illness were significantly and negatively related to step count in all models.

**Table 3:** Standardised Betas (95% CIs) for the models assessing relationships between personality traits and step count (daily average)

	Model 1		Model 2	
	*Β* (95% CI)	*P*	*Β* (95% CI)	*P*
Extraversion	0.08 (− 0.04, 0.20)	.203	0.10 (− 0.02, 0.21)	.104
age	−0.08 (− 0.20, 0.04)	.207	− 0.06 (− 0.18, 0.06)	.326
sex	− 0.01 (− 0.25, 0.24)	.994	− 0.05 (− 0.29, 0.19)	.683
BMI			− 0.28 (− 0.78, − 0.17)	.000
Long-term illness			− 0.47 (0.40, − 0.16)	.000
Agreeableness	0.09 (− 0.04, 0.23)	.167	0.11 (− 0.02, 0.23)	.094
age	−0.07 (− 0.19, 0.06)	.294	− 0.04 (− 0.16, 0.08)	.486
sex	− 0.04 (− 0.31, 0.23)	.780	− 0.09 (− 0.35, 0.17)	.492
BMI			− 0.29 (− 0.41, − 0.17)	.000
Long-term illness			− 0.46 (− 0.77, − 0.16)	.003
Conscientiousness	0.12 (− 0.01, 0.24)	.065	0.08 (− 0.04, 0.19)	.191
age	− 0.07 (− 0.19, 0.06)	.294	− 0.04 (− 0.16, 0.08)	.485
sex	0.03 (− 0.21, 0.29)	.762	− 0.01 (− 0.24, 0.23)	.985
BMI			− 0.27 (− 0.39, − 0.14)	.000
Long-term illness			−0.48 (− 0.78, − 0.18)	.002
Emotional Stability	0.08 (− 0.04, 0.21)	.182	0.06 (− 0.05, 0.18)	.289
age	−0.06 (− 0.19, 0.06)	.310	− 0.05 (− 0.17, 0.07)	.416
sex	0.03 (− 0.21, 0.28)	.793	− 0.02 (− 0.25, 0.22)	.895
BMI			−0.27 (− 0.39, − 0.15)	.000
Long-term illness			−0.45 (− 0.76, − 0.15)	.003
Intellect/Imagination	0.04 (− 0.09, 0.16)	.546	0.07 (− 0.05, 0.18)	.269
age	−0.08 (− 0.20, 0.05)	.224	− 0.05 (− 0.17, 0.07)	.380
sex	0.02 (− 0.23, 0.27)	.852	− 0.02 (− 0.26, 0.22)	.865
BMI			−0.27 (− 0.40, − 0.15)	.000
Long-term illness			−0.48 (− 0.79, − 0.18)	.002
Extraversion	0.08 (− 0.07, 0.22)	.298	0.08 (− 0.06, 0.22)	.252
Agreeableness	0.06 (−0.09, 0.22)	.443	0.07 (−0.07, 0.22)	.320
Conscientiousness	0.09 (−0.04, 0.23)	.170	0.05 (−0.08, 0.18)	.447
Emotional Stability	0.05 (− 0.08, 0.19)	.434	0.04 (−0.09, 0.16)	.583
Intellect/Imagination	−0.05 (− 0.20, 0.10)	.502	− 0.02 (− 0.16, 0.12)	.750
age	− 0.05 (− 0.18, 0.08)	.454	−0.03 (− 0.15, 0.09)	.639
sex	0.01 (−0.27, 0.29)	.966	−0.05 (− 0.32, 0.21)	.688
BMI			−0.28 (− 0.40, − 0.16)	.000
Long-term illness			−0.44 (− 0.75, − 0.13)	.005

*Note.* Model 1 = personality trait + age + sex; Model 2 = Model 1 + BMI + Long-term illness. None of the *p*-values remains significant after False Discovery Rate correction

### Personality traits and total sedentary behaviour

The next set of models examined the associations between five personality traits (entered separately, and together in the final model) and sedentary time, controlling for age and sex (Model 1, Table 4). In the models where one personality trait was entered at a time, higher conscientiousness was significantly associated with less time spent sedentary (*β* = − 0.14, *p* = .025). In the model where all five traits were entered together, extraversion was positively associated with sedentary time (*β* = 0.20, *p* = .005). In the next set of models, additionally controlling for BMI and long-term illness (Model 2), agreeableness was negatively associated with sedentary time both in the one-trait model (*β* = − 0.14, *p* = .029), and in the model where all traits were entered together (*β* = − 0.16, *p* = .033). Furthermore, extraversion was positively associated with sedentary time in the all-traits model (*β* = 0.20, *p* = .004). The effect sizes for all personality traits were similar in Models 1 and 2, regardless of significance levels.

**Table 4:** Standardised Betas (95% CIs) for the models assessing relationships between personality traits and total sedentary time (daily average)

	Model 1		Model 2	
	*Β* (95% CI)	*P*	*Β* (95% CI)	*P*
Extraversion	0.11 (− 0.01, 0.23)	.065	0.10 (− 0.02, 0.21)	.103
age	0.03 (− 0.09 0.15)	.645	0.02 (− 0.09 0.14)	.684
sex	− 0.46 (− 0.70, − 0.22)	.000	− 0.41 (− 0.64, − 0.17)	.000
BMI			0.27 (0.15, 0.39)	.000
Long-term illness			0.11 (−0.19, 0.41)	.481
Agreeableness	−0.11 (− 0.24, 0.02)	.087	− 0.14 (− 0.26, − 0.01)	.029
age	0.03 (− 0.24, 0.02)	.644	0.02 (− 0.10, 0.14)	.703
sex	− 0.35 (− 0.62, − 0.09)	.008	− 0.28 (− 0.54, − 0.03)	.028
BMI			0.29 (0.17, 0.41)	.000
Long-term illness			0.08 (− 0.22, 0.38)	.613
Conscientiousness	−0.14 (− 0.25, − 0.02)	.025	− 0.10 (− 0.22, 0.01)	.075
age	0.01 (− 0.11, 0.14)	.812	0.01 (− 0.11, 0.13)	.886
sex	−0.45 (− 0.69, − 0.21)	.000	−0.40 (− 0.64, − 0.17)	.000
BMI			0.28 (0.15, 0.40)	.000
Long-term illness			0.09 (−0.20, 0.40)	.515
Emotional Stability	0.01 (−0.11, 0.13)	.847	0.01 (− 0.10, 0.13)	.837
age	0.02 (−0.10, 0.14)	.712	0.02 (− 0.10, 0.14)	.722
sex	−0.44 (− 0.68, − 0.20)	.000	−0.39 (− 0.62, − 0.15)	.001
BMI			0.27 (0.15, 0.39)	.000
Long-term illness			0.11 (−0.19, 0.41)	.479
Intellect/Imagination	−0.05 (− 0.17, 0.07)	.400	− 0.07 (− 0.19, 0.04)	.223
age	0.03 (− 0.10, 0.15)	.679	0.02 (− 0.10, 0.14)	.761
sex	−0.43 (− 0.67, − 0.19)	.000	− 0.38 (− 0.61, − 0.14)	.002
BMI			0.28 (0.16, 0.40)	.000
Long-term illness			0.12 (−0.18, 0.42)	.441
Extraversion	0.20 (0.06, 0.34)	.005	0.20 (0.06, 0.33)	.004
Agreeableness	−0.13 (− 0.28, 0.02)	.081	− 0.16 (− 0.30, − 0.01)	.033
Conscientiousness	− 0.11 (− 0.24, 0.02)	.092	−0.07 (− 0.19, 0.06)	.278
Emotional Stability	0.04 (− 0.09, 0.17)	.527	0.04 (−0.08, 0.16)	.535
Intellect/Imagination	−0.08 (− 0.22, 0.06)	.252	− 0.10 (− 0.24, 0.04)	.160
age	0.03 (− 0.10, 0.15)	.653	0.02 (− 0.10, 0.14)	.696
sex	−0.39 (− 0.66, − 0.12)	.005	−0.32 (− 0.58, − 0.06)	.018
BMI			0.28 (0.16, 0.41)	.000
Long-term illness			0.10 (−0.21, 0.40)	.531

*Note.* Model 1 = personality trait(s) + age + sex; Model 2 = Model 1 + BMI + Long-term illness

### Personality traits and sit-to-stand transitions (breaks in sedentary behaviour)

Next, we examined the associations between personality traits and the number of sit-to-stand transitions. In Model 1, controlling for age and sex (Table 5), none of the traits was related to sit-to-stand transitions when they were entered separately. However, extraversion was positively (*β* = 0.16, *p* = .037), and emotional stability negatively (*β* = − 0.13, *p* = .045) associated with sit-to-stand transitions in the models where all five traits were entered together. In the set of models additionally adjusting for BMI and long-term illness (Model 2, Table 5), extraversion was positively associated with sit-to-stand transitions in the one-trait model (*β* = 0.13, *p* = .043), and in the model with all five traits (*β* = 0.16, *p* = .034). No other personality trait was significantly associated with sit-to-stand transitions in fully-adjusted models.

**Table 5:** Standardised Betas (95% CIs) for the models assessing relationships between personality traits and the number of sit-to-stand transitions (daily average)

	Model 1		Model 2	
	*Β* (95% CI)	*P*	*Β* (95% CI)	*P*
Extraversion	0.12 (−0.01, 0.24)	.062	0.13 (0.01, 0.25)	.043
age	−0.06 (− 0.18, 0.06)	.327	− 0.07 (− 0.19, 0.06)	.298
sex	0.00 (− 0.25, 0.25)	.999	− 0.03 (− 0.28, 0.21)	.788
BMI			−0.15 (− 0.28, − 0.03)	.019
Long-term illness			0.07 (−0.25, 0.39)	.655
Agreeableness	−0.04 (− 0.17, 0.10)	.578	− 0.02 (− 0.15, 0.11)	.759
age	− 0.05 (− 0.18, 0.07)	.415	−0.05 (− 0.18, 0.07)	.398
sex	0.07 (−0.20, 0.34)	.603	0.03 (−0.24, 0.30)	.824
BMI			−0.15 (− 0.28, − 0.02)	.022
Long-term illness			0.06 (−0.26, 0.38)	.711
Conscientiousness	0.01 (−0.12, 0.13)	.917	−0.01 (− 0.13, 0.11)	.891
age	−0.05 (− 0.17, 0.08)	.440	− 0.05 (− 0.18, 0.07)	.425
sex	0.03 (− 0.22, 0.28)	.831	− 0.01 (− 0.25, 0.25)	.983
BMI			−0.15 (− 0.28, − 0.02)	.025
Long-term illness			0.05 (−0.27, 0.37)	.760
Emotional Stability	−0.11 (− 0.23, 0.01)	.081	− 0.10 (− 0.23, 0.02)	.096
age	− 0.08 (− 0.21, 0.04)	.203	−0.08 (− 0.21, 0.04)	.190
sex	−0.01 (− 0.25, 0.25)	.985	−0.03 (− 0.28, 0.21)	.795
BMI			−0.14 (− 0.27, − 0.02)	.027
Long-term illness			0.02 (−0.30, 0.35)	.879
Intellect/Imagination	0.05 (−0.07, 0.17)	.423	0.06 (−0.06, 0.18)	.351
age	−0.06 (− 0.18, 0.07)	.353	− 0.06 (− 0.19, 0.06)	.340
sex	0.02 (− 0.23, 0.27)	.870	− 0.01 (− 0.26, 0.24)	.934
BMI			−0.14 (− 0.28, − 0.02)	.023
Long-term illness			0.06 (−0.26, 0.38)	.723
Extraversion	0.16 (0.01, 0.30)	.037	0.16 (0.01, 0.30)	.034
Agreeableness	−0.07 (−0.22, 0.09)	.377	−0.06 (− 0.21, 0.10)	.482
Conscientiousness	0.04 (−0.09, 0.18)	.516	0.02 (−0.11, 0.16)	.739
Emotional Stability	−0.13 (− 0.27, − 0.01)	.045	−0.13 (− 0.26, − 0.01)	.052
Intellect/Imagination	−0.01 (− 0.15, 0.15)	.975	0.01 (− 0.14, 0.15)	.959
age	−0.07 (− 0.19, 0.06)	.309	−0.07 (− 0.19, 0.06)	.317
sex	0.03 (−0.25, 0.31)	.849	−0.01 (− 0.29, 0.27)	.931
BMI			−0.15 (− 0.28, − 0.02)	.025
Long-term illness			−0.01 (− 0.33, 0.32)	.987

*Note.* Model 1 = personality trait + age + sex; Model 2 = Model 1 + BMI + Long-term illness

### Correction for multiple testing

We used the false discovery rate procedure to correct for the large number of dependent tests we ran. After doing this, none of the *p*-values reached the conventional thresholds of significance (all *p*s > .07). We, therefore, conclude that all the above-presented associations between personality traits and measures of sedentary behaviour could be considered Type I errors.

## Discussion

The present study examined whether personality traits are cross-sectionally associated with device-based measures of physical activity and sedentary behaviour in older age. The main measure of physical activity was the average number of steps taken per day. The results of this study showed that none of the personality traits was significantly associated with step count. The main measure of total sedentary behaviour was the average percent of waking time spent sedentary. Conscientiousness was negatively associated with sedentary time when entered alone, but this association did not remain after controlling for BMI and long-term illness, nor when other personality traits were entered in the model. Extraversion was positively associated with sedentary time both in the model controlling for age and sex, and in the fully adjusted model, but only when all five traits were entered together. Finally, agreeableness was negatively associated with sedentary time both alone and in the all-trait model, but only after adjusting for BMI and long-term illness. An additional measure of the pattern of sedentary behaviour was the average number of sit-to-stand transitions. Extraversion was positively associated with the number of sit-to-stand transitions in the all-trait model controlling for age and sex, and both alone and in the all-trait fully-adjusted models. Emotional stability was negatively associated with sit-to-stand transitions in the all-trait model controlling for age and sex, but this association did not remain after additionally adjusting for BMI and long-term illness. Finally, after correcting for multiple comparisons, none of the associations between personality traits and device-based measures of physical activity/sedentary behaviour remained significant.

Our study extends previous literature that examined the associations between personality traits and physical activity. However, our null results with respect to step count are inconsistent with a number of previous reports on the personality-physical activity associations, based on self-reported and device-based measures of physical activity. On the other hand, our null results with respect to device-based measures of total sedentary behaviour are consistent with those previously reported in a smaller sample of older adults [20]. The association of the pattern of accumulation of sedentary behaviour with personality traits has not been previously reported. The consistencies and inconsistencies are described in more detail below.

The associations between conscientiousness and a wide-range of health behaviours, including physical activity, are well-established [30]. This is true for both self-reported [14] and device-based measures of physical activitity [20]. However, no such association was found in the present study. Similarly, our findings do not replicate those that found that higher extraversion is associated with higher levels of physical activity both in a series of studies utilising self-reported measures of physical activity, [14] and in a recent study using device-based measures of physical activity [20]. While the outcome measures and covariates were the same, our current study is conducted in a larger sample with 80% power to detect an effect size of 0.17 [32], which is in the range reported in previous studies. Another difference is that participants in our sample were less physically active than those in the study conducted by Artese et al. (6940 average steps/day in our sample versus 8832 in the previous study).

The findings of the present study showed that there is no association between openess to experience and physical activity, while a recent meta-analysis showed a positive association between openness to experience and physical activity [14]. The null results in the present study are, however, in line with a recent study that used device-based measures [20]. It is possible that people higher in openness are more likely to report being more physically active, even when they are not when measured using devices such as accelerometers. A similar pattern is seen for cognitive ability, where higher intelligence is found to be associated with higher levels of self-reported physical activity, [33, 34] a finding currently not replicated using device-based measures of physical activity/sedentary behaviour [35].

A previous study using device-based measures of physical activity found that higher agreeableness is related to being more physically active as indexed by step count [20]. However, a meta-analysis of studies that mostly used self-reported measures of physical activity in all ages found no such effect [14]. Artese et al. suggest that the link between agreeableness and activity may be specific to older populations, due to individuals higher in agreeableness being more compassionate and more likely to help others, and in older populations both having more time to do so (having more free time in retirement), and also knowing more people with functional limitations and chronic illness that might need help [20]. While this idea is corroborated by our recent finding that retired older adults who engage in caring behaviours spend less time in sedentary activities [36] the null results of the present study do not provide further support for this idea, at least at the trait level of personality.

There was no evidence of a relationship between emotional stability (neuroticism) and device-based measures of physical activity or sedentary behaviour, which was in contrast to findings of previous studies reporting that higher levels of neuroticism (lower emotional stability) are associated with being less physically active [19, 20]. However, one study found that a group of women, but not men, lowest in neuroticism performed fewest steps in a day [18]. Inconsistent findings with respect to neuroticism have been found for a range of health outcomes, including type 2 diabetes, [37] and all-cause mortality [38]. There are some indications that the associations between neuroticism and health may be moderated by other variables, including the personality trait conscientiousness [39], and self-rated health [40]. It could be suggested that inconsistent associations between neuroticism and health outcomes may be in part due to its inconsistent association with health behaviours, such as physical activity in older age. Further research is needed to understand the reason for these inconsistencies.

In addition, the current study explored the association of personality traits with the pattern of accumulation of sedentary behaviour. There is evidence of some beneficial metabolic effects (e.g. glycemia) from experimental studies of the acute effects of regular breaks in sedentary behaviour of at least light physical activity and from observational studies that a larger number of device-based measured breaks in sedentary behaviour was associated with reduced obesity [41]. However, the current study did not find any association of the five-factor model’s personality traits with the number of device-based breaks in sedentary behaviour. Our study is in line with previously reported lack of associations between personality and device-based measures of sedentary behaviour [20].

We have shown in a large group of community dwelling men and women aged approximately 79 in the United Kingdom, that personality traits were not key factors in whether people engage in total sedentary behaviour or how they break up their sedentary behaviour, after adjustment for age, gender, weight status and general health. Determinants of sedentary behaviour can be used to target interventions aiming to reduce and break up pronged sedentary behaviour. Personality traits represent substantially stable traits and are not ideal themselves as intervention targets. However, behaviours associated with personality traits might be. Moreover, understanding how personality traits interact with sedentary behaviour can help tailor intervention content and delivery. As other determinants of sedentary behaviour have been shown to be important (such as socio-economic deprivation), these represent more appropriate targets for public health interventions.

### Strengths and limitations

The biggest strength of the present study is the use of device-based measures of physical activity/sedentary behaviour, which minimises possible self-report bias. Additionally, we used a monitor that measures postural sitting, as opposed to low hip movement to assess sedentary behaviour. However, there are no available algorithms to reliably identify moderate intensity physical activity in older adults using the monitor, and we were unable to investigate the association of personality with physical activity intensity alongside the association with total physical activity (represented by step count). The study sample was larger than those used in previous studies of personality and device-based measures of physical activity, thus having more statistical power. Participants were all born in the same year, and lived in the United Kingdom, which may limit generalisability of the results. Additionally, measures of personality and walking/sedentary behaviour were taken at the same time point, so it was impossible to examine whether there are any prospective associations between personality and physical activity and sedentary behaviour.

## Conclusions

In conclusion, no evidence was found that personality is related to device-based measures of physical activity, total sedentary behaviour or breaks in sedentary behaviour (pattern of accumulation) in older age. However, due to inconsistencies with previous studies that focused on physical activity, further replications are needed before any firm conclusions are made about the associations between personality and physical activity/sedentary behaviour in older age.

## Data Availability

The dataset used in this study is available upon request due to ethical restrictions on open data sharing. The consent forms for the study included that participants’ data, some of which is sensitive, would only be used for the purposes of research. This is ensured by restricting access to the data, which is available through submitting a data access form to i.deary@ed.ac.uk or lbc1936@ed.ac.uk.

## Abbreviations

FFM: Five factor model
IPIP: International personality item pool
LBC1936: Lothian Birth Cohort 1936
MET: Metabolic equivalent
N: Number
